# Return to Sport, Re-injury and Performance After the Anterior Cruciate Ligament Reconstruction: Evaluating the Role of International Knee Documentation Committee (IKDC) and Knee Injury and Osteoarthritis Outcome Score (KOOS) Scoring Systems

**DOI:** 10.7759/cureus.58574

**Published:** 2024-04-19

**Authors:** Andrea Noah Paris, Colin NG, Thomas Gatt, Nicole Galdes, Hussein Wehbi, Duncan Marston, Denise Galdes, Nikita Carabott, Ivan Esposito

**Affiliations:** 1 Surgery, Mater Dei Hospital, Msida, MLT; 2 Orthopaedics and Trauma, Mater Dei Hospital, Msida, MLT; 3 Internal Medicine, Mater Dei Hospital, Msida, MLT; 4 Medicine and Surgery, University of Malta, Msida, MLT

**Keywords:** performance, return to sport, re-injury, koos score, ikdc score, anterior cruciate ligament (acl) reconstruction

## Abstract

Introduction

Anterior cruciate ligament (ACL) tears are common injuries that can considerably impact an individual’s quality of life and athletic performance. In these cases, surgical reconstruction of the ligament can be considered to restore stability to the knee. This study aims to investigate the time taken for individuals to return to sport post-ACL reconstruction, assess the rate of re-injury and evaluate the reliability of the International Knee Documentation Committee (IKDC) and Knee Injury and Osteoarthritis Outcome Score (KOOS) scoring systems in predicting a return to sport at the pre-injury level.

Methods

In this retrospective study, a total of 104 patients who underwent ACL reconstruction between January 2016 and December 2022 by one surgical team at Mater Dei Hospital, Malta were evaluated using a self-administered questionnaire. The questionnaire was based on different components including the sport practised at the time of injury, sport engagement classification, return to sport, the ability to return to pre-injury levels of performance and re-injury. The participants then had to fill in IKDC and KOOS evaluation forms.

Results

In this study, 73% (n=76) of individuals successfully returned to sport after ACL reconstruction, with no significant difference being found between professional and recreational athletes (Chi-squared=0.00455, p=0.95). After reconstruction, 31.7% (n=33) of participants experienced an ipsilateral or contralateral ACL tear, with those returning to sport within six months showing a fivefold increase in re-injury risk compared to individuals who returned at eight or 12 months, suggesting a significant association between return duration and re-injury. The relationship between scoring systems and return to sport at the pre-injury level of performance was analysed using binary logistic regression, revealing that achieving scores of 85.6 or higher in IKDC or 89 or higher in KOOS meant having a 95% probability of returning to sport at the pre-injury level.

Conclusions

By considering these scoring systems with other post-operative criteria, clinicians can offer a more customised rehabilitation plan tailored to each patient who undergoes ACL reconstruction.

## Introduction

Anterior cruciate ligament (ACL) injuries represent a prevalent occurrence among individuals engaged in regular sports activities. In the United Kingdom alone, an estimated 15,000 individuals undergo ACL reconstruction surgery annually [1]. Despite the frequency of these procedures, the outcomes of ACL reconstruction surgeries remain an area of ongoing investigation as outcomes often do not meet the desired expectations [2].

This study aims to investigate the time it takes for individuals to resume sporting activities after surgery, as well as to assess the rate of subsequent re-injury to ipsilateral or contra-lateral ACL. Additionally, we seek to determine whether the International Knee Documentation Committee (IKDC) or Knee Injury and Osteoarthritis Outcome Score (KOOS) scoring systems can reliably predict the likelihood of returning to sports at the same performance level as before the injury [3]. Specifically, we aim to determine if obtaining a certain score in either IKDC or KOOS is strongly associated with a successful return to sports and the restoration of previous performance levels.

## Materials and methods

Data was retrospectively collected on all patients who underwent primary ACL reconstruction surgery between January 2016 and December 2022, performed by a single team at Mater Dei Hospital, Malta. All patients received bone-patellar tendon-bone (BPTB) or Ligament Augmentation and Reconstruction System (LARS) grafts.

A structured questionnaire was formulated to comprehensively assess the following components: the sport practised when injury was sustained, classification of sports engagement into either professional or recreational, the duration taken to resume sporting activities post-surgery, the ability to return to pre-injury levels of performance and occurrences of subsequent ACL injuries. In addition to the primary questionnaire, participants were also provided with IKDC and KOOS evaluation forms. The questionnaire, along with the attached evaluation forms, was distributed retrospectively to patients via electronic mail.

The study implemented the following exclusion criteria: patients who had undergone ACL reconstruction using hamstrings or quadriceps tendon grafts and individuals who already had a primary ACL reconstruction and underwent a secondary ACL reconstruction for a re-injury in the ipsilateral knee or a new injury in the contralateral knee with an intact ACL graft on the opposite knee.

Approval was obtained from the Mater Dei Data Protection Office and the Trauma and Orthopaedics Clinical Chairperson. Data collected was inputted into the Statistical Package for the Social Sciences (IBM SPSS Statistics for Windows, IBM Corp., Version 28.0, Armonk, NY). Chi-squared testing was used to assess for significance between categorical variables. Pearson’s correlation coefficient was utilized to assess the significance of the relationship between the distribution of KOOS and IKDC scores. Logistic regression analysis was employed to predict or determine a cut-off value for returning to sport and pre-injury performance based on IKDC/KOOS scores. A significance threshold of p<0.05 was established to denote statistical significance.

The IKDC is a subjective scale designed to assess patients' overall knee function, covering three main categories: symptoms, sports activity and knee function. Scores range from 1 to 100, with higher scores indicating better knee function [4].

The KOOS on the other hand is a specialized tool designed to gauge patients' perspectives on their knee health and related issues. It assesses both short-term and long-term impacts of knee injuries, comprising 42 items across five distinct areas: pain, symptoms beyond pain, daily functioning, physical activity and sport participation, and quality of life related to the knee. Scores range from 1 to 100, with higher scores indicating better knee function [5].

Recreational level sport was defined as participation for enjoyment, leisure and personal well-being rather than for payment or competitive purposes [6]. Professional-level sport was defined as either receiving some form of payment for their involvement or competing at national or international levels. The definition of "professional sport" was adjusted to better align with the context of Malta, a small country where athletes typically do not earn the majority of their income from sports. Traditionally, "professional sport" is defined as individuals who derive over 50% of their income from sports-related activities [7]. However, recognising the unique circumstances of Malta's sporting landscape, this definition was modified to reflect a broader range of athletes who engage in sports at a competitive level, even if they do not primarily rely on it as their main source of income.

## Results

Demographics

**Figure 1:**
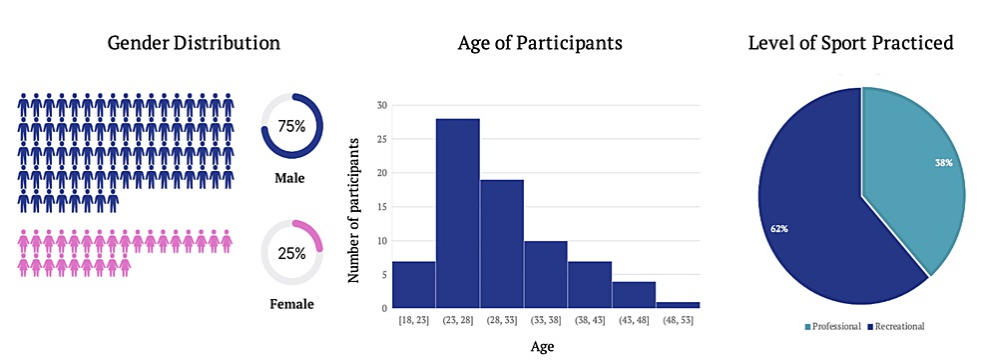
Demographics

Of the 330 patients contacted, a total of 104 completed the questionnaire (31.5% response rate), 78 being male (75%) and 26 being female (25%) (Figure 1). The mean age of participants at the time of surgery was 26 years (SD=7.06). The majority of individuals (n=64, 61.5%) engaged in sports at a recreational level while the remaining (n=40, 38.5%) participated in sports professionally.

**Figure 2:**
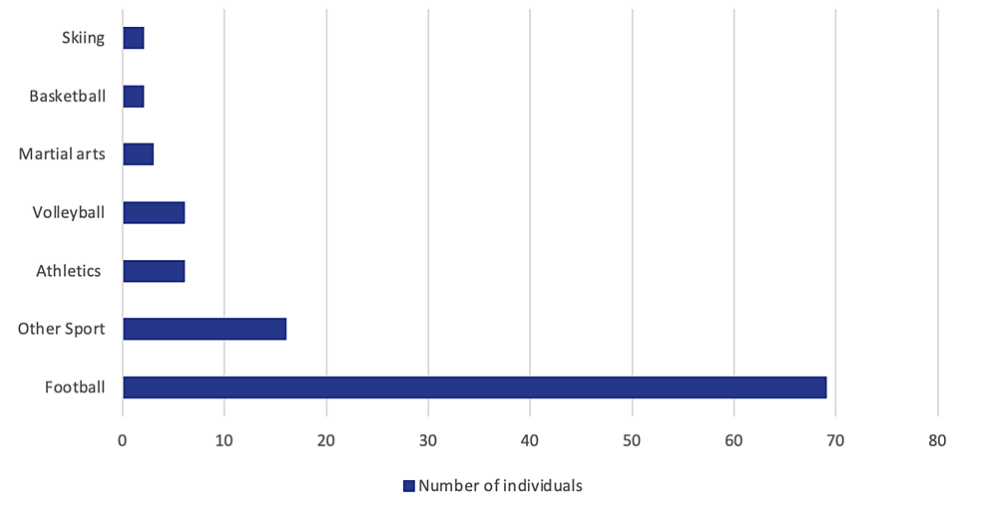
Sport Practised at the Time of Injury

The most common sport practised at the time of the primary injury was football (n=69, 66.3%), followed by volleyball (n=6, 5.8%) and athletics (n=6, 5.8%). Other sports (n=16, 15.4%) included activities where only a single participant engaged in the specific sport, which encompassed disciplines such as rugby, tennis, kickboxing, dancing, and more (Figure 2). Among the participants, most underwent a BPTB graft procedure (n=75, 72.1%), while the remaining participants received a LARS graft (n=29, 27.9%).

Return to sport

**Figure 3:**
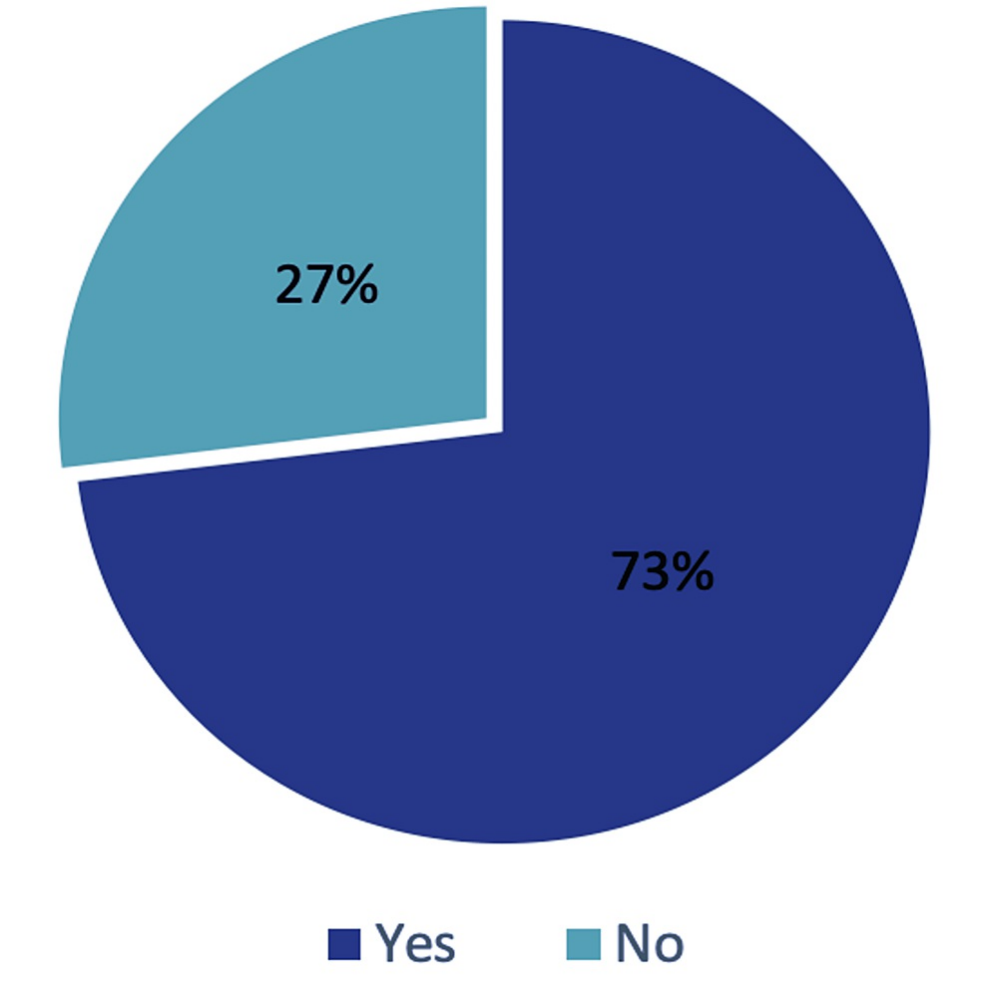
Ability to Return to Sport

Most individuals (n=76, 73.1%) successfully returned to sports, while the rest (n=28, 26.9%) were unable to resume sporting activities (Figure 3).

**Figure 4:**
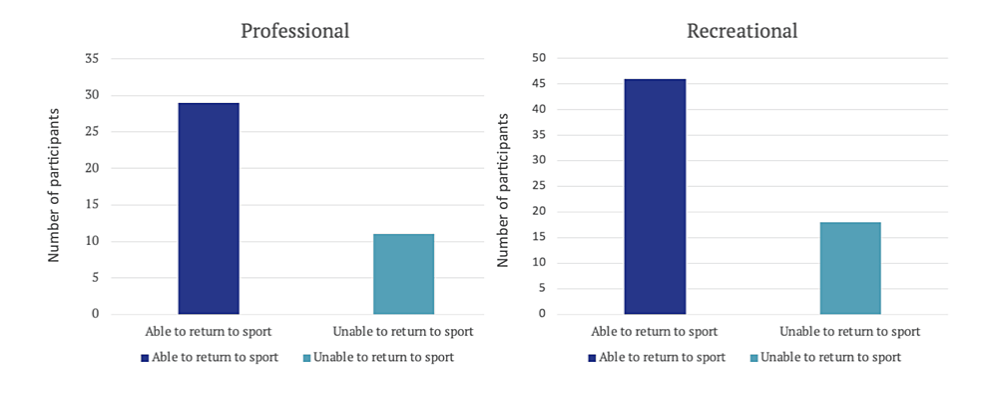
Ability to Return to Sport in Professional vs Recreational Athletes

Professional athletes demonstrated a marginally higher return to sport rate (n=29, 72.5%) than recreational athletes (n=46, 71.9%) (Figure 4). Using the Chi-squared test X^2^ (1, N=104) = 0.00455 with a p-value=0.95, it was demonstrated there was no significant difference in return to sport between professional and recreational athletes.

Primary reasons hindering return to sport in this study included factors such as knee instability, pain and psychological barriers.

Re-injury rate

**Figure 5:**
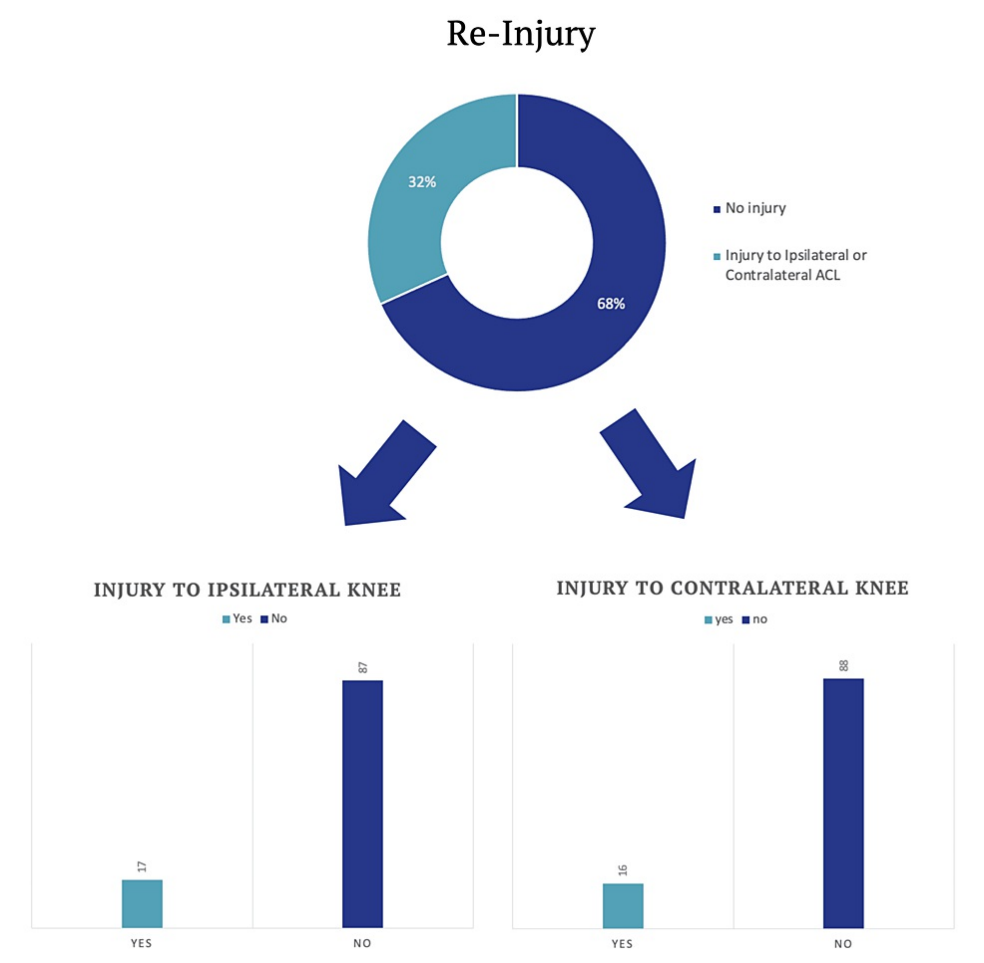
Re-injury to Ipsilateral and Contralateral ACL ACL: anterior cruciate ligament

In this study, 31.7% (n=33) of individuals experienced either an ipsilateral or contralateral ACL tear post-surgery. On further assessment of these injuries, it was revealed that 16.3% (n=17) of individuals suffered an ipsilateral ACL injury while 15.3% (n=16) experienced a new injury to the contralateral ACL (Figure 5).

**Figure 6:**
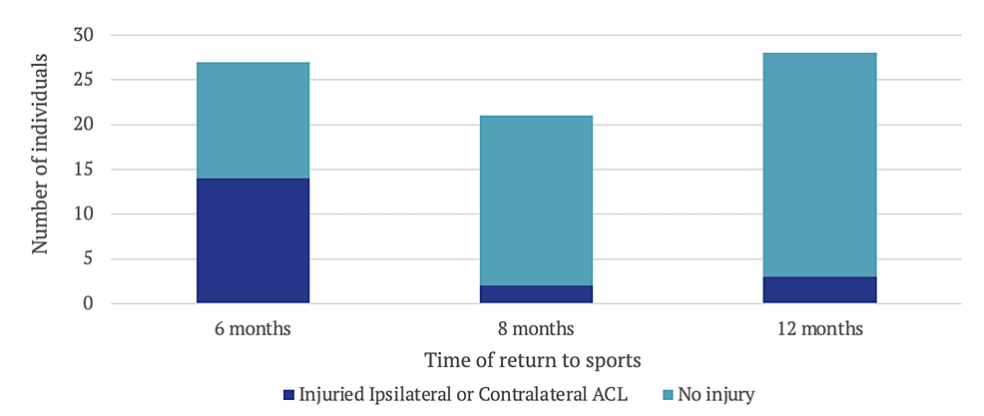
Time of Return to Sport vs Re-injury ACL: anterior cruciate ligament

The study also examined the relationship between the rate of re-injury and the timing of return to sport. Participants were classified based on when they resumed sporting activities: at six months, eight months, or 12 months post-surgery. A total of 51.9% (n=14) of individuals who returned to sport at six months experienced an injury to either the ipsilateral or contralateral ACL. In contrast, participants returning at eight months and 12 months had re-injury rates of 9.5% (n=2) and 10.7% (n=3) respectively (Figure 6). This suggests that an early return to sport at six months was associated with a fivefold increase in the risk of re-injuring the ACL at two years or more after surgery, whether on the ipsilateral or contralateral side.

Performance

**Figure 7:**
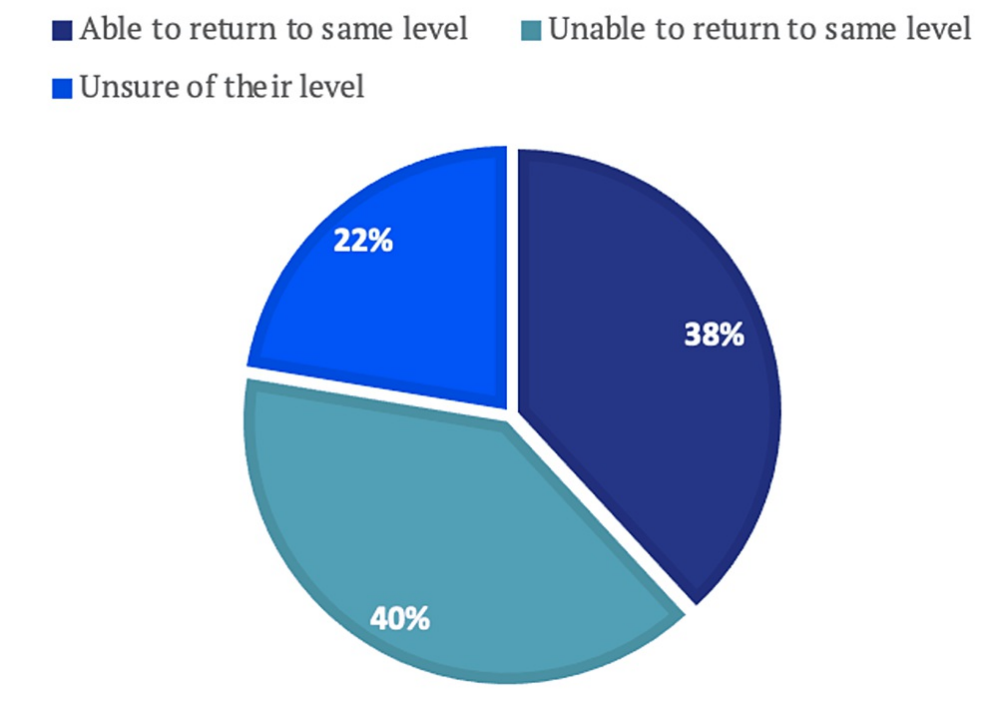
Ability to Return to Pre-injury Level of Performance

Performance was assessed subjectively by asking participants about their perception of achieving pre-injury performance levels following surgery and rehabilitation. Out of the respondents, 38.2% (n=29) expressed confidence in having regained their former performance level while 39.5% (n=30) reported feeling unable to reach their previous performance standards. An additional 22% (n=17) of participants remained uncertain whether they reached pre-injury levels (Figure 7).

KOOS and IKDC scores

**Figure 8:**
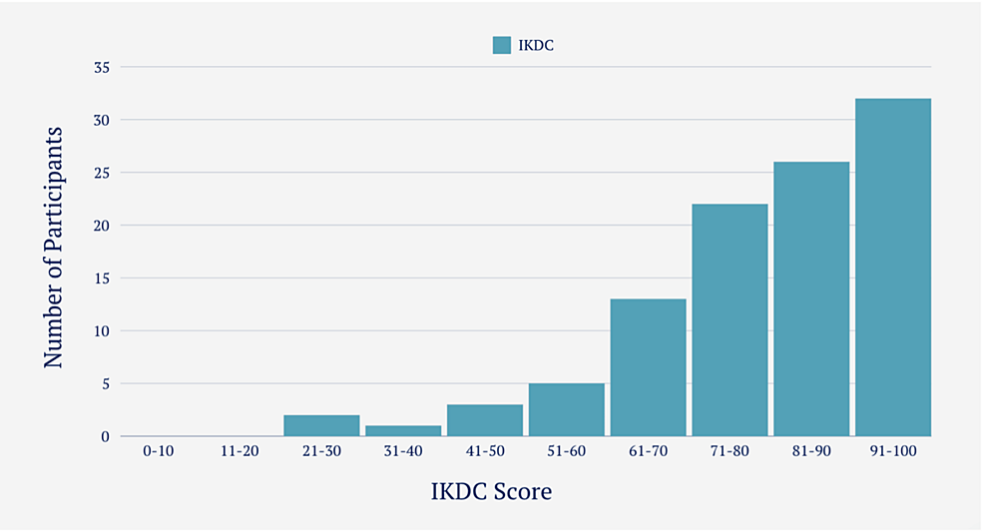
IKDC Scoring Distribution Amongst Participants IKDC: International Knee Documentation Committee

**Figure 9:**
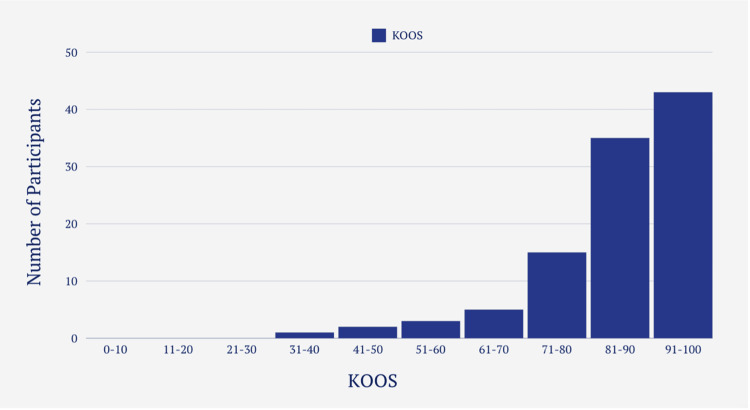
KOOS Scoring Distribution Amongst Participants KOOS: Knee Injury and Osteoarthritis Outcome Score

The mean IKDC score for participants able to return to sport was 84.6 (SD=13.79), compared to 69.9 (SD=18.33) for those unable to return (Figure 8). Similarly, the mean KOOS for returners was 88 (SD=11), while non-returners scored 76 (SD=16) (Figure 9). Participants tended to achieve slightly higher scores on the KOOS measure. Using Pearson’s correlation coefficient (r=0.868, p<0.01) there was a significant large positive relationship between the distribution of KOOS and the distribution of IKDC scores.

Return to sport cut-off using IKDC and KOOS

**Figure 10:**
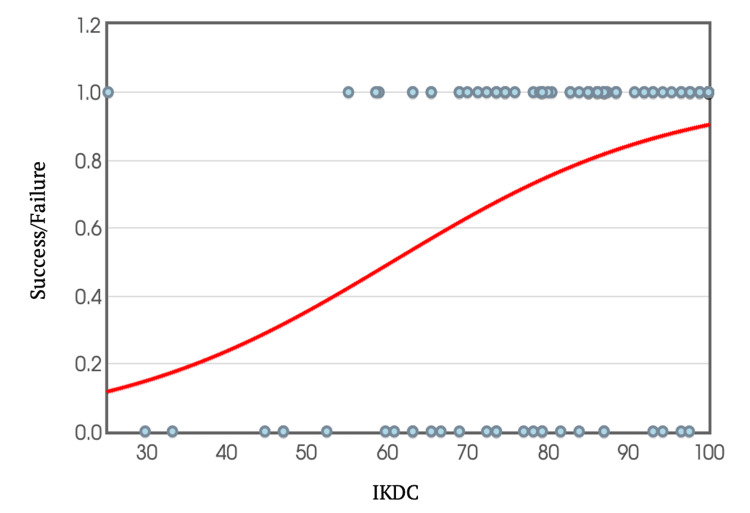
IKDC Score Plotted Against Success/Failure Rate in Return to Sport IKDC: International Knee Documentation Committee

**Figure 11:**
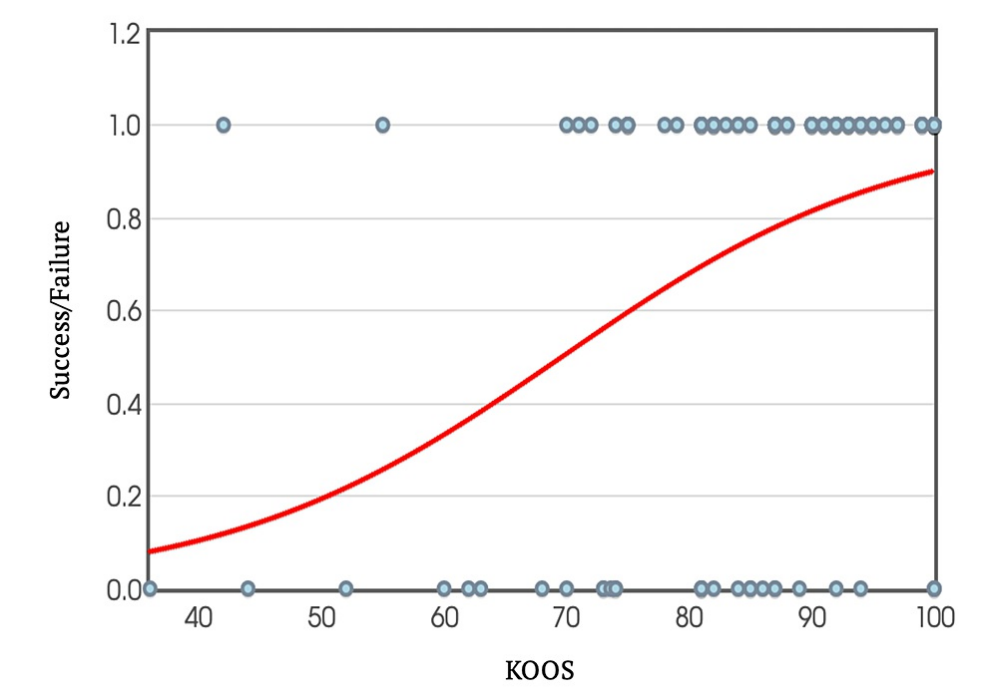
KOOS Plotted Against Success/Failure Rate in Return to Sport KOOS: Knee Injury and Osteoarthritis Outcome Score

To further analyse the relationship between scores and return to sport, binary logistic regression equations were used to create graphs, plotting individual IKDC and KOOS scores against the ability or inability to return to sport (Figures 10-11). Extrapolating from these graphs, a score above 60.6 in the IKDC and 69.6 in KOOS predicts the likelihood of returning to sport at a 95% confidence interval.

IKDC and KOOS return to pre-injury performance cut-off

**Figure 12:**
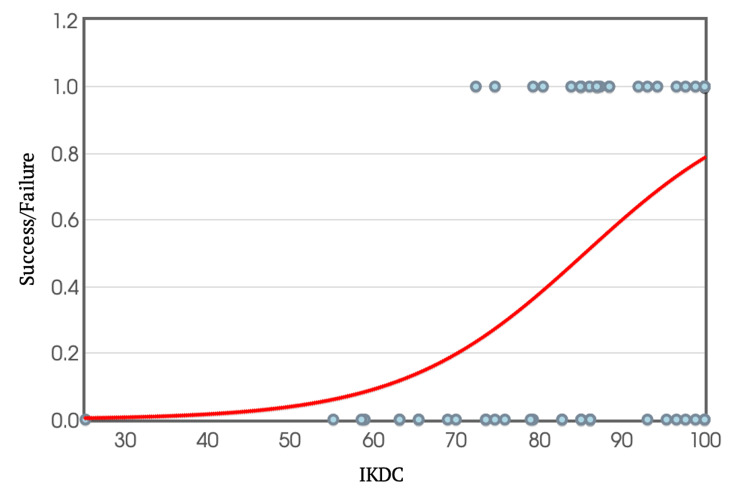
IKDC Scores Against Ability to Return to Sport at Pre-injury Level of Performance IKDC: International Knee Documentation Committee

**Figure 13:**
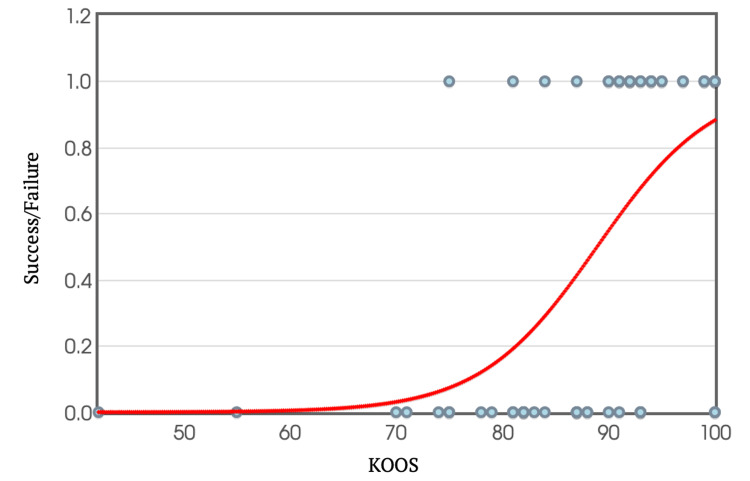
KOOS Against Ability to Return to Sport at Pre-injury Level of Performance KOOS: Knee Injury and Osteoarthritis Outcome Score

Again, using binary logistic regression equations, individual IKDC and KOOS scores were plotted against their ability to return to pre-injury performance levels (Figures 12-13). Extrapolating from these graphs, it was determined that scoring above 85.6 in the IKDC and 88.9 in KOOS was required to achieve a pre-injury performance level at a 95% confidence interval.

## Discussion

Return to sport

In this study, 73% of individuals successfully returned to their sport. There was no significant difference (p<0.05) in return to sport rates between professional (72.5%) and recreational athletes (71.9%).

A systematic review by Ardern et al. [8] discussed and compared studies on return to sport post-ACL reconstruction, demonstrating an overall return to sport rate of 82%, with 63% achieving a return to sport at pre-injury level performance. While this study showed a slightly lower return to sport rate (73%), a more significant disparity was noted with the return to pre-injury levels, where only 38% of individuals were able to do so.

It is worth noting that in the questionnaire distributed an "unsure" option was included, and 22% of respondents selected this choice. This may have introduced response bias, because some unsure individuals might have been more likely to answer "yes" if the "unsure" option was not given. Additionally, the KOOS and IKDC scores of these unsure individuals were very similar to those who returned to their previous level of performance. Therefore, we believe that the observed lower percentage of individuals returning to pre-injury levels could have been influenced by this factor.

Re-injury

The overall re-injury rate for both ipsilateral and contra-lateral ACL of 32% observed in this study, encompassing both ipsilateral and contralateral ACL tears, falls within the range reported in existing literature [9]. In an article by Paterno et al. [10] comparing multiple studies, re-injury rates ranging from 6% to 31% were observed. Although our study's re-injury rate was on the higher end of this spectrum, several factors, including the timing of early return to sport, likely contributed to this outcome as discussed further on.

Timing of return to sport and re-injury

There are currently two prevailing approaches to returning to sport after ACL injury: an accelerated approach, advocating return at six months, and a conservative approach, advising return at 9-12 months [11]. The benefits of early return to sport at six months are evident, leading to significant reductions in disability, pain, and depression, along with improvements in overall quality of life [12]. However, these advantages must be carefully weighed against the risk of graft or contralateral ACL re-injury, as indicated in this study. At the six-month mark, our cohort displayed a concerning 51.9% re-injury rate, indicating that half of the individuals who returned to the sport at six months experienced either a re-tear of the same ACL or a new tear in the opposite ACL.

Conversely, returning to sport at the 12-month mark showed a notably lower re-injury rate of 10.7% for both the ipsilateral and contralateral ACL. Interestingly, the re-injury rate at eight months mirrored that of 12 months at 9.5%, suggesting a potential balance point for encouraging return to sport.

This finding aligns with observations made by Meredith et al. [13], indicating a 51% reduction in re-injury incidence for each month that return to sport is delayed between months five and nine postoperatively. This may relate to graft maturation and adequate bone healing before resuming strenuous physical activity [14].

In this study, the focus was solely on comparing the timing of individuals' return to sport. However, it's essential to recognise that the decision to return to sport involves multiple factors [15]. As demonstrated by various criteria such as range of motion, presence of effusion, quadriceps and hamstring strength, joint laxity, limb symmetry index, IKDC, KOOS, single limb hop tests, balance, and others, a comprehensive assessment is necessary before clearing an individual to return to sport [16].

Nonetheless, the timing of eight months offers a potential balance between the conservative and accelerated methods. This timeframe allows for the potential benefits of both approaches to be realised.

IKDC and KOOS score

The distribution pattern of IKDC and KOOS exhibited remarkable similarity (Figures 8-9). In fact, using Pearson’s correlation coefficient, a substantial and positive correlation was observed between the two. This suggests that individuals who achieved high scores in the IKDC also tended to score high in the KOOS and vice versa. The utilization of two separate subjective scoring systems served as a safeguard measure to ensure that individuals with favorable scores indeed reflected genuine outcomes, rather than random chance.

IKDC Score

In a study conducted by Lentz et al. [12], an IKDC score cut-off of greater than 93 was utilized as one of the criteria for determining return to sport. In another study performed by Toole et al. [16], an IKDC score of greater than 90 was used in conjunction with strength measures (quadriceps and hamstrings) and single leg hop testing achieving a Limb Symmetry Index (LSI) of 90% or greater as criteria to return to sport.

In this study, the IKDC score was taken as the sole criterion and aimed to identify an IKDC score value where our cohort, at a 95% confidence interval, successfully returned to the sport, as well as identifying a score cut-off for returning to their previous level of performance. Those who managed to return to some level of sport had a mean IKDC score of 84.6, whereas those who did not return scored a mean of 69.9.

Upon further analysis using logistic regression and extrapolating data from it, scoring an IKDC of more than 60.6 meant having a 95% chance of returning to some form of sport. Although this IKDC score may seem surprisingly low, it is essential to note that it encompasses any form of return to sport.

When considering performance specifically, individuals who were able to return to the same level of performance had a mean IKDC score of 90.7. Further analysis using logistic regression revealed that scoring above 85.6 on the IKDC scale meant having a 95% chance of returning to the pre-injury level of sport. Therefore, it is suggested that a slightly lower IKDC criterion of 85.6 can be used as a predictor/criterion for return to pre-injury level of performance.

KOOS Score

Among participants who were able to return to sport, the mean KOOS was 88, while those unable to return achieved a mean of 76. In this study, 75% of participants achieved KOOS scores in the range of 81-100 (Figure 9). Comparatively, in a study conducted by Bley et al. [17], assessing KOOS 12 months post-ACL reconstruction surgery, 55% of their cohort scored within the same range. One possible explanation for the higher scores observed in our cohort could be attributed to the timing of our assessments. Our participants were requested to complete the questionnaire two years or more post-surgery, allowing for an extended period of rehabilitation. This extended duration may have contributed to improved knee function and higher KOOS scores compared to those assessed at the 12-month mark in the study by Bley et al.

**Figure 14:**
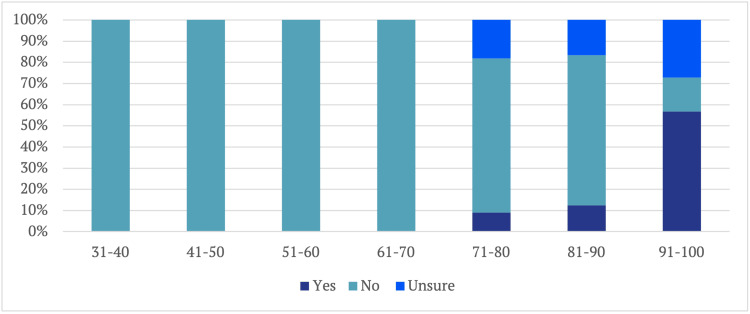
Performance Level in Relation to KOOS Score KOOS: Knee Injury and Osteoarthritis Outcome Score

Examining KOOS in more detail revealed interesting patterns. Of those scoring between 91 and 100, 55% were able to return to the same level of performance. Conversely, only about 12% of individuals scoring 81-90 on the KOOS were able to do so, and a mere 9% returned to the sport with scores falling between 71 and 80. Notably, no individuals scoring less than 70% on the KOOS were able to return to the same level of performance (Figure 14).

Like the IKDC score analysis, a cut-off criterion for KOOS was established using logistic regression. It was found that a KOOS of greater than 70 at a 95% confidence interval was required for return to sport, while a score of 89 was necessary to achieve a 95% confidence interval for returning to the same level of performance.

Strengths and limitations

While this study offers important insights into returning to sport post-ACL reconstruction, it is important to acknowledge both its strengths and limitations.

A substantial strength of this research is its novelty, especially in the local context, as there is a lacuna in data in Malta. Therefore, it can act as a guide both in clinical decision-making as well as in future research studies. Another strength is that this study considered all patients who underwent BTPB and LARS grafts in Malta within the six-year period.

Some limitations also exist in the study that should be taken into consideration. Firstly, it is the nature of data collection, which was done retrospectively through a questionnaire. This may have introduced recall bias or inaccuracy, especially if the participant cannot remember past events correctly. Another limitation to note is that no pre-operative IKDC/KOOS values were available for post-operative comparison. Additionally, no participants who underwent hamstring tendon graft reconstruction for ACL were considered. This might have introduced sampling bias, making the sample less representative of the population.

Furthermore, all the ACL reconstruction surgeries were performed by a single surgical team. While this may have introduced operator bias it is worth noting that it is the only team that performed BTPB and LARS graft ACL reconstructions during the study period.

## Conclusions

Our study yielded similar findings regarding both the return to sport and re-injury rates when compared to prior studies conducted with similar methodologies.

Returning to sport at the six-month mark was linked to a fivefold increase in the risk of re-injuring the ipsilateral or contralateral ACL. However, our study suggests that returning to sport at eight months may serve as a transitional period between the accelerated and conservative approaches, potentially allowing individuals to gain the benefits of both strategies.

Although IKDC and KOOS scoring systems are not typically used as the sole criteria for determining clearance to return to sport following ACL reconstruction, establishing cut-offs of 86 for IKDC and 89 for KOOS can provide valuable guidance to clinicians. While these measures alone may not carry significant weight, integrating them with other criteria can assist in effectively categorizing ACL reconstruction patients in the postoperative rehabilitation period. This holistic approach enables clinicians to make more informed decisions and tailor rehabilitation plans to meet the unique needs of individual patients.
